# Demonstration of 50 Gbps Long-Haul D-Band Radio-over-Fiber System with 2D-Convolutional Neural Network Equalizer for Joint Phase Noise and Nonlinearity Mitigation

**DOI:** 10.3390/s25123661

**Published:** 2025-06-11

**Authors:** Yachen Jiang, Sicong Xu, Qihang Wang, Jie Zhang, Jingtao Ge, Jingwen Lin, Yuan Ma, Siqi Wang, Zhihang Ou, Wen Zhou

**Affiliations:** Key Laboratory for Information Science of Electromagnetic Waves (MoE), Shanghai Institute for Advanced Communication and Data Science, Fudan University, Shanghai 200433, China; 21307130299@m.fudan.edu.cn (Y.J.); 24110720169@m.fudan.edu.cn (S.X.); 23110720122@m.fudan.edu.cn (Q.W.); 23210720305@m.fudan.edu.cn (J.Z.); 23210720159@m.fudan.edu.cn (J.G.); 23210720058@m.fudan.edu.cn (J.L.); 22210720195@m.fudan.edu.cn (Y.M.); 24210720273@m.fudan.edu.cn (S.W.); 24210720069@m.fudan.edu.cn (Z.O.)

**Keywords:** 2D-convolutional neural network, D-band, photonics-aided, wireless transmission, nonlinear equalization, phase noise compensation

## Abstract

High demand for 6G wireless has made photonics-aided D-band (110–170 GHz) communication a research priority. Photonics-aided technology integrates optical and wireless communications to boost spectral efficiency and transmission distance. This study presents a Radio-over-Fiber (RoF) communication system utilizing photonics-aided technology for 4600 m long-distance D-band transmission. We successfully show the transmission of a 50 Gbps (25 Gbaud) QPSK signal utilizing a 128.75 GHz carrier frequency. Notwithstanding these encouraging outcomes, RoF systems encounter considerable obstacles, including pronounced nonlinear distortions and phase noise related to laser linewidth. Numerous factors can induce nonlinear impairments, including high-power amplifiers (PAs) in wireless channels, the operational mechanisms of optoelectronic devices (such as electrical amplifiers, modulators, and photodiodes), and elevated optical power levels during fiber transmission. Phase noise (PN) is generated by laser linewidth. Despite the notable advantages of classical Volterra series and deep neural network (DNN) methods in alleviating nonlinear distortion, they display considerable performance limitations in adjusting for phase noise. To address these problems, we propose a novel post-processing approach utilizing a two-dimensional convolutional neural network (2D-CNN). This methodology allows for the extraction of intricate features from data preprocessed using traditional Digital Signal Processing (DSP) techniques, enabling concurrent compensation for phase noise and nonlinear distortions. The 4600 m long-distance D-band transmission experiment demonstrated that the proposed 2D-CNN post-processing method achieved a Bit Error Rate (BER) of 5.3 × 10^−3^ at 8 dBm optical power, satisfying the soft-decision forward error correction (SD-FEC) criterion of 1.56 × 10^−2^ with a 15% overhead. The 2D-CNN outperformed Volterra series and deep neural network approaches in long-haul D-band RoF systems by compensating for phase noise and nonlinear distortions via spatiotemporal feature integration, hierarchical feature extraction, and nonlinear modelling.

## 1. Introduction

The fast progression of worldwide informatization is instigating significant modifications in mobile communication technology. With the advent of the 6G communication era, burgeoning applications like the metaverse, autonomous driving, and holographic communication necessitate wireless networks characterized by ultra-high data speeds (about 100 Gbps), ultra-low latency (sub-millisecond), and ultra-large connection density [1]. The conventional microwave frequency ranges are becoming progressively insufficient for these demands. In this context, the D-band (110–170 GHz) has emerged as a highly attractive contender for 6G wireless communication. The enormous bandwidth of the D-band facilitates substantial data transmission, offering the capability for high-rate communications [2].

Photonics-aided millimeter-wave generation uses optical heterodyne beating and optical frequency comb generation to overcome bandwidth, transmission loss, and phase stability issues in conventional electronic devices at the D-band (110–170 GHz) [3]. This method uses photonic techniques to achieve high spectral purity, low phase noise, and wide bandwidth scalability, enabling the reliable generation of high-frequency millimeter-wave signals in the demanding D-band frequency range [4].

RoF technology integrates the high-bandwidth, low-loss attributes of optical communication with the adaptability of wireless communication [5,6,7]. In a RoF system, radio-frequency (RF) signals are modulated onto optical carriers, transmitted over optical fibers, and subsequently converted back to RF signals at the receiver. This method capitalizes on the low-loss characteristics of optical fibers to efficiently increase signal transmission distance and employs the high-bandwidth benefits of optical communication to improve system transmission capacity [8,9,10,11].

The nonlinear properties of optoelectronic devices, such as modulators and photodetectors, generate supplementary harmonic components during signal modulation and detection, disrupting the original signal. The Kerr effect in fiber transmission generates nonlinear phase noise by rendering the optical signal’s phase contingent upon its intensity. These effects collectively result in amplitude distortion and phase noise, significantly impairing system performance. The restricted linewidth of lasers presents another challenge: while laser output is not a perfect monochromatic signal but encompasses a specific frequency range (i.e., linewidth), it results in random phase drift. As transmission distance and signal rate escalate, the accumulation of phase drift causes rotation and diffusion of the constellation diagram of received signals, degrading signal quality and elevating the bit error rate [12].

In D-band RoF systems, addressing nonlinear distortion and phase noise compensation is a significant research problem. Classical nonlinear approaches like the Volterra series are based on linear time-invariant (LTI) system theory. These methods estimate nonlinear distortion using polynomial models to demonstrate how power amplifiers (PAs) link input and output as polynomial terms and delayed input signals. Limitations include the fast growth of model complexity as nonlinear order rises, which requires more processing power and hardware. In real-world application, high-order model parameters are hard to estimate, fit too tightly to training data, and perform poorly in varied conditions, resulting in inaccurate compensation performance.

Due to their robust nonlinear fitting characteristics, deep neural networks (DNNs) have seen extensive use in nonlinear compensation in recent years. They can autonomously acquire complex signal feature patterns to mitigate nonlinear distortion [13,14]. Nevertheless, DNNs have significant constraints in phase noise compensation. Phase noise is a random process that changes over time, making it hard for traditional statistical learning methods to effectively understand its changing characteristics. Furthermore, phase noise and nonlinear distortion demonstrate a coupling effect; nonetheless, current methods frequently treat them separately, overlooking their interrelations. This oversight results in diminished compensation efficiency and inadequate enhancement of system performance.

Two-dimensional convolutional neural networks (2D-CNNs) have been very successful in areas like image processing and computer vision, showing strong abilities to identify features over time and space [15]. Their basic structure includes convolutional layers, pooling layers, and fully connected layers, which help in effectively handling grid-like data and finding patterns and relationships in the information. Research teams have successfully adapted 2D-CNNs for wireless communication, leading to better results in improving signal quality, lowering bit error rates (BER), and increasing data capacity [16,17]. This work offers novel insights for concurrently tackling nonlinear distortion and phase noise in D-band RoF systems. The 2D-CNN can learn the spatial correlations and distribution characteristics of phase noise in signals by adaptively updating weight parameters, dynamically adjusting convolutional kernel weights during backpropagation optimization to effectively model and suppress phase noise. However, putting this into practice faces challenges, such as creating the right network designs and settings that fit the system’s specific issues, as well as choosing the right training data and methods to improve performance and reliability when channel conditions change and device parameters vary. In recent years, a series of research achievements have been made in the field of terahertz signal transmission, and the relevant progress is shown in Table 1 [18,19,20,21,22,23].

This research presents a 2D-CNN post-processing technique grounded in spatiotemporal feature fusion. In a 4600 m experiment using a single-channel photonics-assisted terahertz wireless transmission, the traditional Digital Signal Processing (DSP) technique was first used, and then the new 2D-CNN algorithm was applied to improve the signal. The experiments showed that this new method reduced nonlinear noise better and lowered the bit error rate (BER) compared to traditional DSP algorithms, along with a detailed look at the results. A D-band RoF system using photonics-assisted technology was created, allowing for stable transmission of QPSK signals at a speed of 25 Gbaud (which is 50 Gbps) over a distance of 4600 m at a frequency of 128.75 GHz. The system used a method to create millimeter-wave signals: it takes electrical signals, changes them into optical signals with a Mach–Zehnder modulator (MZM), boosts them with a PA, converts them into high-frequency millimeter-wave signals with a photodetector (PD), and then sends them wirelessly using strong directional antennas. Experimental findings indicate that our photonics-aided millimeter-wave generating method enabled steady transmission of a 50 Gbps (25 Gbaud) QPSK signal across a distance of 4600 m at 128.75 GHz, confirming its viability for long-distance communications in the D-band. In a transmission scenario with an optical power of 8 dBm, the proposed method diminished the system BER to 5.3 × 10^−3^, markedly surpassing the traditional Volterra series (BER = 1.4 × 10^−2^) and DNN approaches (BER = 1.3 × 10^−2^) and remaining below the SD-FEC threshold of 1.56 × 10^−2^.

## 2. Principle

### 2.1. Phase Recovery Theory

In RoF systems, phase noise caused by laser linewidth generates intricate interference. Conventional phase recovery algorithms encounter difficulties in accurately extracting and compensating for phase fluctuations [24]. Neural networks characterize phase noise in the channel by training on extensive datasets and exhibiting distinct benefits [25,26].

The QPSK symbol sequence produced by the transmitter constitutes a time-domain signal. The width of a laser’s light beam causes the signal’s phase to shift over time, resulting in random changes in the phase that can be considered a sequence of offsets $\left\{\theta \left(n\right)\right\}$. Mathematically, we represent the received signal as complex exponential modulation:

(1) $$y(n)=x(n)\cdot e^{j\theta \left(n\right)}*h\left(n\right)+n\left(n\right)$$

where $\theta \left(n\right)$ represents the phase noise of the *n*-th symbol, ∗ denotes the convolution operation, $h\left(n\right)$ is the channel impulse response, and $n\left(n\right)$ is additive Gaussian white noise.

**Figure 1:**
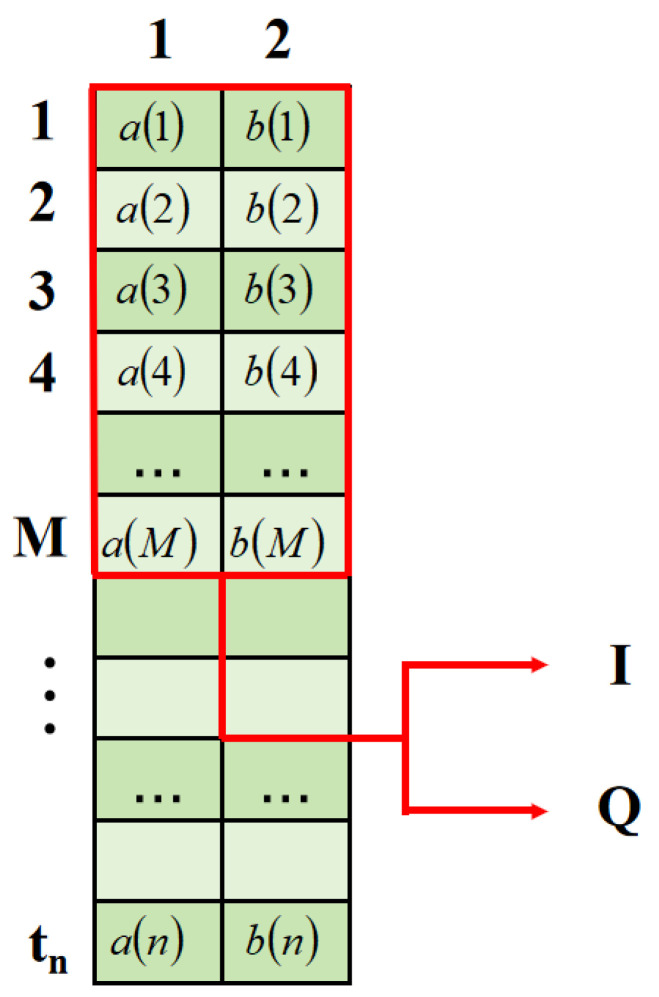
Two-dimensional structure of the input data.

By decomposing the time-domain received signal into its real and imaginary components, we formulate a two-dimensional input tensor:

(2) $$Input=\left[\begin{array}{c} a\left(n\right)\\ b\left(n\right) \end{array}\right]\in R^{M\times 2}$$

where $a\left(n\right)$ and $b\left(n\right)$ denote the real and imaginary components, M signifies the duration of the time frame, and the two channels correspond to the real-part and imaginary-part channels. Figure 1 demonstrates the two-dimensional structure of the input data. Continuous symbol segments are retrieved using a sliding window that encompasses the current symbol and $\frac{M-1}{2}$ neighboring symbols on either side.

Each row of the input tensor shows the real and imaginary parts of a single symbol, and each column relates to the real or imaginary parts of nearby symbols, forming a two-dimensional feature matrix that helps with 2D convolution extraction. The temporal direction refers to the phase continuity of neighboring symbols, whereas the channel direction pertains to the amplitude-phase mapping connection between the real and imaginary components.

Under optimal circumstances, the real and imaginary components of the channel fulfil $a^{2}+b^{2}=1$. In the event of a phase deflection θ, according to Euler’s formula $e^{j\theta }=\cos\theta +j\sin\theta$, the signal is expressed as:

(3) $$\;\left(a+jb\right)\left(\cos\theta +j\sin\theta \right)=a\cos\theta -b\sin\theta +j\left(a\sin\theta +b\cos\theta \right)$$

The new real component $a^{\prime }=a\cos\theta -b\sin\theta$ and the imaginary component $b^{\prime }=a\sin\theta +b\cos\theta$ are derived. Post-normalization, the total number of squares remains equal to 1; however, the phase has altered. Through extensive data training, the convolution kernel can recognize the “abnormal” coupling pattern induced by phase rotation. The convolution kernel weights will enhance the response to this pattern, thereby extracting the attributes of phase rotation.

Phase correction is analogous to doing a complex conjugate rotation on the incoming signal:

(4) $$\hat{y}\left(n\right)=y\left(n\right)\cdot e^{-j\hat{\theta }\left(n\right)}=\left(a\left(n\right)+jb\left(n\right)\right)\left(\cos\hat{\theta }\left(n\right)-j\sin\hat{\theta }\left(n\right)\right)$$

Upon expansion, the real component is $\hat{a}\left(n\right)=a\left(n\right)\cos\hat{\theta }\left(n\right)+b\left(n\right)\sin\hat{\theta }\left(n\right)$, while the imaginary component is $\hat{b}\left(n\right)=-a\left(n\right)\sin\hat{\theta }\left(n\right)+b\left(n\right)\cos\hat{\theta }\left(n\right)$. The 2D-CNN does the inverse transformation of phase deflection by acquiring the weights of the convolution kernel.

The mathematical representation of the convolution operation is:

(5) Yl=ReLUXl∗Kl+Bl

Here, $X^{\left(l\right)}$ denotes the output of the *l*-th convolutional layer, $K^{\left(l\right)}$ signifies the convolution kernel, and $B^{\left(l\right)}$ indicates the bias. ReLU is an activation function that imparts nonlinear properties, allowing the network to learn intricate phase-mapping correlations. The mean squared error (MSE) serves as the loss function, defined as the average squared discrepancy between the real and imaginary components of the recovered signal and the original signal:

(6) $$L=\frac{1}{2M}\sum_{m=1}^{M}\left(||{\hat{s}}^{m}-s^{m}|{|}_{2}^{2}\right)+\lambda \sum_{\theta }||\theta |{|}_{1}$$

**Figure 2:**
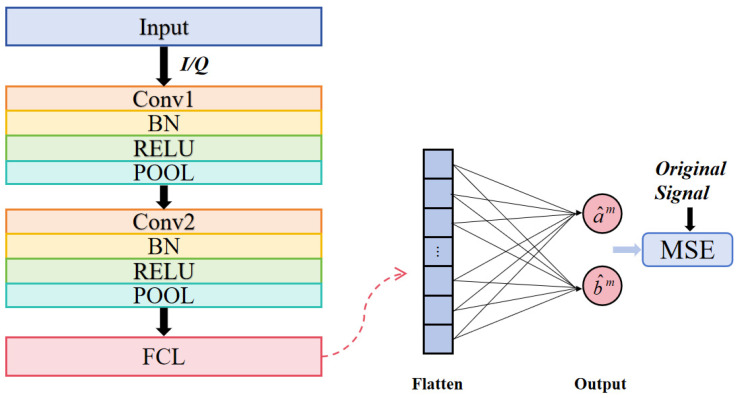
Schematic flowchart of 2D-CNN.

Let M denote the batch size, ${\hat{s}}^{m}=\left[{\hat{a}}^{m},{\hat{b}}^{m}\right]$ represent the reconstructed signal, $s^{m}=\left[a^{m},b^{m}\right]$ signify the original signal, and λ indicate the $L_{1}$ regularization coefficient. Figure 2 shows the flowchart of 2D-CNN. The network has convolutional, batch normalization, activation, pooling, and fully connected layers. The initial and subsequent convolutional layers are succeeded by a batch normalization layer (BN) to standardize feature maps, mitigate internal covariate shift, and accelerate training convergence. RELU introduces non-linearity, enabling the network to develop intricate patterns. POOL layers downsample features by max-pooling, decreasing dimensionality while preserving essential information.

Subsequent to the convolutional and pooling stages, the feature maps are transformed into a one-dimensional vector and input into a fully connected layer (FCL), which integrates the accumulated spatial-temporal characteristics to reconstruct the signal. The FCL transforms high-dimensional feature space into output space, enabling the network to generate final predictions or indicate adjustments. This architecture enables the 2D-CNN to integrate spatiotemporal data, therefore diminishing phase noise and nonlinear distortions.

In backpropagation, the gradient of the loss function concerning the convolution kernel $\frac{\partial L}{\partial {Κ}^{\left(l\right)}}$ is computed using the following steps:

The gradient of the loss concerning the output feature map is:

(7) $$\frac{\partial L}{\partial Y^{\left(l\right)}}=\delta^{\left(l+1\right)}*K^{\left(l+1\right)}$$

Here, $\delta^{\left(l\right)}$ denotes the error value of the *l*-th layer. $\delta^{\left(l+1\right)}=\frac{\partial L}{\partial Y^{\left(l+1\right)}}=\hat{s}-s$, representing the error value of the subsequent layer. The derivative of the activation function concerning the linear transformation is:

(8) $$\frac{\partial Y^{\left(l\right)}}{\partial (X^{\left(l\right)}*K^{\left(l\right)})}={\mathrm{ReLU}}^{\prime }(Y^{\left(l\right)})=I(Y^{\left(l\right)}>0)$$

Here, I(⋅) denotes an indicator function that equals 1 when the input exceeds 0 and 0 otherwise. The gradient of the feature map concerning the convolution kernel is:

(9) $$\frac{\partial (X^{\left(l\right)}*K^{\left(l\right)})}{\partial K^{\left(l\right)}}=X^{\left(l\right)}$$

Consolidated using the chain rule:

(10) $$\frac{\partial L}{\partial K^{\left(l\right)}}=\frac{\partial L}{\partial Y^{\left(l\right)}}⊙\frac{\partial Y^{\left(l\right)}}{\partial (X^{\left(l\right)}*K^{\left(l\right)})}*\frac{\partial (X^{\left(l\right)}*K^{\left(l\right)})}{\partial K^{\left(l\right)}}$$

Here, ⊙ denotes the element-wise product (Hadamard product), where corresponding elements of two matrices are multiplied together. Deriving the formula for error term propagation:

(11) $$\delta^{\left(l\right)}=\left(\delta^{\left(l+1\right)}*K^{\left(l+1\right)}\right)⊙\sigma^{\prime }\left(Y^{\left(l\right)}\right)$$

(12) $$\frac{\partial L}{\partial K^{\left(l\right)}}=\sum_{i=1}^{N}\delta_{i}^{\left(l\right)}*X_{i}^{\left(l\right)}+\lambda \cdot \mathrm{sign}(K^{\left(l\right)})$$

Here, $sign\left(\cdot \right)$ indicates the sign function. The forward propagation of the fully linked layer is as follows:

(13) $$\hat{s}=W_{fc}\cdot \mathrm{flatten}(Y^{\left(L\right)})+b_{fc}$$

where $\hat{s}=\left[\begin{array}{c} \hat{a}(n)\\ \hat{b}(n) \end{array}\right]$. The gradient of the loss concerning the output is:

(14) $$\frac{\partial L}{\partial \hat{s}}=\frac{1}{N}(\hat{s}-s)$$

The gradient of the output concerning the input of the fully connected layer is:

(15) $$\frac{\partial \hat{s}}{\partial \mathrm{flatten}(Y^{\left(L\right)})}=W_{fc}^{T}$$

The gradient of the input to the fully connected layer concerning the weight is:

(16) $$\frac{\partial \mathrm{flatten}(Y^{\left(L\right)})}{\partial W_{fc}}=\mathrm{flatten}{\left(Y^{\left(L\right)}\right)}^{T}$$

Consolidated using the chain rule:

(17) $$\frac{\partial L}{\partial W_{fc}}=\frac{\partial L}{\partial \hat{s}}\cdot \frac{\partial \hat{s}}{\partial \mathrm{flatten}(Y^{\left(L\right)})}\cdot \frac{\partial \mathrm{flatten}(Y^{\left(L\right)})}{\partial W_{fc}}$$

Ultimately, we derive:

(18) $$\frac{\partial L}{\partial W_{fc}}=\frac{1}{N}(\hat{s}-s)\cdot \mathrm{flatten}{\left(Y^{\left(L\right)}\right)}^{T}+\lambda \cdot \mathrm{sign}(W_{fc})$$

The gradient of the bias $b_{fc}$ is expressed as:

(19) $$\frac{\partial L}{\partial b_{fc}}=\frac{\partial L}{\partial \hat{s}}\cdot \frac{\partial \hat{s}}{\partial b_{fc}}=\frac{1}{N}(\hat{s}-s)$$

The parameter update formula using the AdamW optimizer, which separates weight decay from gradient computation, is as follows:

Estimation of gradient momentum and variance:

(20) $$m_{t}=\beta_{1}m_{t-1}+(1-\beta_{1})g_{t}$$

(21) $$v_{t}=\beta_{2}v_{t-1}+(1-\beta_{2})g_{t}^{2}$$

(22) $${\hat{m}}_{t}=\frac{m_{t}}{1-\beta_{1}^{t}},\;{\hat{v}}_{t}=\frac{v_{t}}{1-\beta_{2}^{t}}$$

Here, $g_{t}$ represents the current gradient, whereas $\beta_{1}=0.9$ and $\beta_{2}=0.999$ denote the momentum coefficients. The parameter update including weight decay is expressed as:

(23) $$\theta_{t}=\theta_{t-1}-\eta \left(\frac{{\hat{m}}_{t}}{\sqrt{{\hat{v}}_{t}}+\epsilon }+\lambda \theta_{t-1}\right)$$

Here, η denotes the learning rate, λ signifies the weight-decay coefficient, $\varepsilon =10^{-8}$ represents the smoothing term, and θ encompasses parameters such as $K^{\left(l\right)}$, $W_{fc}$, and $b_{fc}$, among others. Through the optimization of weights via backpropagation and the minimization of the MSE, a comprehensive mapping from the noisy signal to the denoised output post-neural network processing is accomplished.

(24) $$f_{CNN}\left(\left[\begin{array}{c} a\left(n\right)\\ b\left(n\right) \end{array}\right];W\right)=\left[\begin{array}{cc} \cos\hat{\theta }\left(n\right) & \sin\hat{\theta }\left(n\right)\\ -\sin\hat{\theta }\left(n\right) & \cos\hat{\theta }\left(n\right) \end{array}\right]\left[\begin{array}{c} a\left(n\right)\\ b\left(n\right) \end{array}\right]=\left[\begin{array}{c} \hat{a}\left(n\right)\\ \hat{b}\left(n\right) \end{array}\right]$$

The network outputs $\hat{a}\left(n\right)$ and $\hat{b}\left(n\right)$ constitute the reconstructed complex signal $\hat{y}\left(n\right)=\hat{a}\left(n\right)+j\hat{b}\left(n\right)$.

### 2.2. Principle of Two-Dimensional Convolutional Neural Networks

A 2D-CNN is a deep learning model, mainly made up of convolutional layers, pooling layers, and fully connected layers [27]. The convolutional layer utilizes the feature maps from the preceding layer as input for convolution processes, subsequently generating the output feature map via an activation function [28,29]. The output feature map can be represented as:

(25) $$x_{n}^{l}=f(\sum_{k=1}^{K}x_{k}^{l-1}*W_{nk}^{l}+b_{n}^{l})$$

Here, $W_{\mathrm{nk}}^{l}$ denotes the kernel connecting the *n*-th and *k*-th features of the *l*-th layer, while $x_{n}^{l}$ and $x_{k}^{l-1}$ signify the *n*-th and *k*-th feature maps of the *l*-th and (*l* − 1)-th layers, respectively. Additionally, $b_{n}^{l}$ represents the associated bias.

In executing intricate convolution processes, we typically split them into real-valued operations. The 2D-CNN works on the real and imaginary parts of the complex signal separately before combining them, which allows it to perform the convolution operation on the complex signal. Activation functions are crucial in neural networks because they introduce nonlinear properties to the network. After a linear transformation in the convolutional layer, the results are mixed with a nonlinear activation function before moving on to the next layer. In 2D-CNN, there are two common types of activation functions: traditional S-type functions like sigmoid and tanh, and non-saturated functions like ReLU, Leaky ReLU, ELU, PReLU, and RReLU [30]. However, traditional S-type functions can cause the problem of vanishing gradients as the model gets deeper. However, with traditional S-type functions, the problem of vanishing gradients starts to occur as the model gets deeper. Non-saturated activation functions have been proposed to address this issue [31,32].

The ReLU function is predominantly utilized in convolutional layers. The mathematical representation of ReLU is as follows:

(26) ReLUx={x,x<0x,x≥0=max0,x

When *x* is non-negative, ReLU outputs the input, whereas if *x* is negative, it outputs 0. This simple process gives the ReLU function nonlinear features and remarkable computing efficiency. Sigmoid functional representation:

(27) $$Sigmoid(x)=\frac{1}{1+e^{-x}}$$

The range is [0, 1], making it ideal for model output functions that generate probability values inside (0, 1). This method works for models that produce projected probabilities, such as binary classification categories or confidence levels. However, above a certain range, this function’s derivative approaches 0. This will cause neurons to become saturated during training, slowing weight updates during backpropagation and complicating model training. We call this the vanishing gradient issue. The tanh function formula is:

(28) $$\tanh(x)=\frac{e^{x}-e^{-x}}{e^{x}+e^{-x}}$$

The hidden layer uses the tanh function, whereas the output layer uses the sigmoid function in normal binary classification tasks. In 2019, Diganta Misra announced the Mish activation function [33]. This research used it:

(29) $$Mish\left(x\right)=x\tanh\left(\ln\left(1+e^{x}\right)\right)$$

Mish accepts negative inputs and prevents ReLU from rejecting negative gradients using ReLU’s straight component and tanh’s curves. Mish is smoother and has fewer vanishing gradients than ReLU. Dropout [34], a regularization technique for neural network training, mitigated this issue in the 2D-CNN. Dropout randomly deactivates neurons with a probability *p*, resulting in an output of 0. Neuron output is maintained throughout training and testing by retaining and scaling all neurons.

A loss function quantifies the discrepancy between expected and real labels. The loss function modifies the parameters of network training. Changing model parameters lowers training loss function. The primary metric is MSE:

(30) $$MSE=\frac{1}{n}{\sum_{i=1}^{n}\left(y_{i}-{\hat{y}}_{i}\right)}^{2}$$

In this formula, $y_{i}$ is the actual value, ${\hat{y}}_{i}$ is the predicted value, and n is the total number of samples. Cross-entropy loss measures how different two probability distributions are and is often used in classification tasks, especially when using the softmax activation function to create probability distributions. In the context of binary classification problems:

(31) $$L=-\frac{1}{n}\sum_{i=1}^{n}\left[y_{i}\log\left({\hat{y}}_{i}\right)+\left(1-y_{i}\right)\log\left(1-{\hat{y}}_{i}\right)\right]$$

For multi-classification problems:

(32) $$L=-\frac{1}{n}\sum_{i=1}^{n}\sum_{j=1}^{C}y_{ij}\log\left({\hat{y}}_{ij}\right)$$

where *C* denotes the number of categories. We assessed mean squared error and cross-entropy loss during the training process. The MSE quantifies the average squared deviation between predicted and actual values, thereby assessing the model’s predictive capability for signal recovery and continuous signal values. If signal recovery, continuous signal value forecasting, or constellation diagram production is unnecessary, using cross-entropy as the loss function may enhance classification outcomes and reduce bit-error rates.

We evaluated three optimizers throughout the training process: stochastic gradient descent (SGD), Adam, and AdamW [35]. Optimizers adjust parameters autonomously according to rules derived from gradients generated by backpropagation, which reflect changes in loss associated with the parameters. SGD, the fundamental optimizer, iteratively adjusts model parameters with mini-batch gradients. The formula is:

(33) $$\theta_{t+1}=\theta_{t}-\eta \nabla_{\theta_{t}}L\left(\theta_{t}\right)$$

The advantages include straightforward implementation, little processing burden, limited hyperparameters, and effective performance with small datasets or uncomplicated models. The learning rate dictates the speed of learning and may result in entrapment inside local minima, rendering the process unstable.

Adam modifies the learning rate via momentum by averaging gradients and their squares. The approach accelerates convergence in complex data distributions, exhibits insensitivity to the initial learning rate, and requires few parameter adjustments. Adam employs more memory and may encounter convergence issues. AdamW, an enhanced version of Adam, updates parameters without gradient computation and incorporates weight decay for regularization. This technique enhances model generalization and stability by removing the effects of momentum and adaptive learning rates on classic Adam’s L2 regularization. The automatic modification of the learning rate and weight-decay coefficient mitigates overfitting.

This study carefully looks at different neural network algorithms and traditional DSP algorithms through organized experiments; measures how things like model complexity, optimizers, and activation functions affect phase-recovery performance; and finds the best model design and settings for the D-band RoF system in these situations.

## 3. Experimental Setup

**Figure 3:**
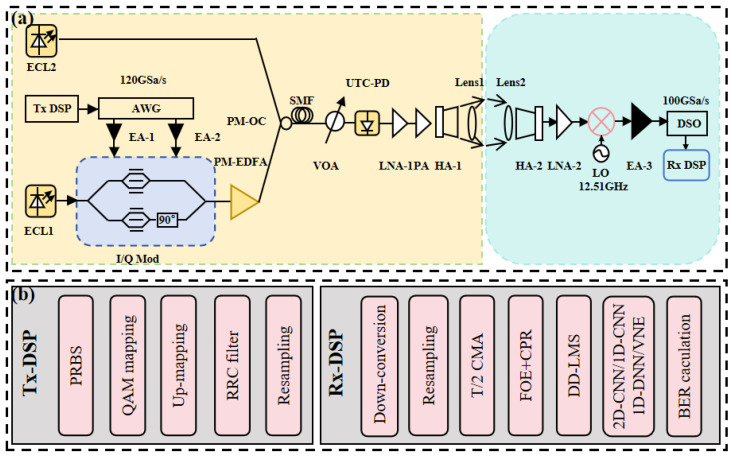
The experimental setup: (**a**) single-channel 4600 m wireless testing configuration, (**b**) transmitter-side and receiver-side DSP.

In this experiment, we built a single-channel experimental setup for long-distance wireless communication in the D-band using photonics-assisted technologies. The transmitter and receiver were situated on two campuses of Fudan University. The wireless transmitter was positioned atop Guanghua Building in Handan Campus, which stands at 142 m, and the wireless receiver was situated on the top floor of the Physics Building, at a 4600 m distance from the transmitter and at a height of 24 m. The system architecture is depicted in Figure 3a.

Figure 3b illustrates the distinct procedures pertaining to the transmitter DSP (Tx DSP) and receiver DSP (Rx DSP). The baseband signal was created offline in MATLAB (R2023b) on the transmitter side. The bit sequence was first mapped to QPSK signals. Subsequent to up-sampling, the signals underwent root-raised cosine (RRC) pulse shaping with a roll-off value of 0.01. Next, we produced the digital signal by resampling and input it into a 120 GSa/s arbitrary waveform generator (AWG), resulting in a 25 Gbaud QPSK signal. The signals were enhanced by two parallel electrical amplifiers (EAs), each possessing a gain of 25 dB.

Two free-running external-cavity lasers (ECLs) were utilized in the experiment. ECL-1 possessed a wavelength of 1550.00 nm, a linewidth under 100 kHz, and an output power of 12 dBm; ECL-2 had a wavelength of 1551.08 nm, with identical linewidth and output power as ECL-1. The steady light from ECL-1 was adjusted by an I/Q modulator that had a 3 dB bandwidth of 30 GHz, using the 12 dBm optical carrier from ECL-1 to complete the optical modulation. The modulated signal was boosted to 12 dBm by polarization maintaining erbium-doped fiber amplifier (PM-EDFA), and then it was combined with optical carrier from ECL-2 by polarization maintaining optical combiner (PM-OC). The combined signal travelled through 100 m of Single-Mode Fiber-28, and then its optical power was adjusted by a variable optical attenuator (VOA) before it went into a uni-traveling carrier photodiode (UTC-PD). In the UTC-PD, a D-band millimeter-wave signal was produced using optical heterodyne beating. Due to the quick attenuation of the signal during free-space transmission, amplification was necessary. Cascaded LNA-1, possessing a gain of 18 dB, and PA, exhibiting a saturated output power of 13 dBm, were employed to facilitate 4600 m wireless transmission. A transmitting horn antenna (HA-1) with a gain of 25 dB emitted the enhanced signal into free space. To improve the directivity and transmission range of the signal, a pair of lenses was positioned in front of the transmitting and receiving antennas, respectively. Lens 1 possessed a diameter of 10 cm, whereas Lens 2 had a diameter of 60 cm.

At the receiver end, the signal was initially amplified by LNA-2 with a gain of 33 dB. The 12.51 GHz local oscillator (LO) was then increased in strength by 12 times in the mixer, mixed with the terahertz signal, and changed into an intermediate-frequency (IF) signal. The down-converted IF signal was then boosted by EA-3, which increased its strength by 26 dB, and was finally recorded by a digital storage oscilloscope (DSO) that sampled at 100 GSa/s and had a 3 dB bandwidth of 33 GHz.

Figure 3b illustrates that the IF signal acquired at the receiver was processed offline using DSP in MATLAB. Initially, down-conversion and resampling were executed to transform the IF signal into a baseband signal. A 31-tap T/2 constant modulus algorithm (CMA) was used to improve the signal quality and reduce interference caused by the way the channel transmits data. Next, frequency-offset estimation (FOE) was done to reduce the effects of frequency drift caused by the laser’s frequency offset, and carrier-phase recovery (CPR) was performed to improve the phase noise caused by the laser’s linewidth. A 51-tap direct-detection least-mean-square (DD-LMS) technique was employed for equalization.

After using traditional DSP algorithms, the resulting signal was further equalized with a 2D-CNN, a 1D-CNN, a 1D-DNN, and a Volterra nonlinear equalizer (VNE). The VNE used signal-processing methods to reduce distortion in the signal caused by device nonlinearity. The 1D-DNN and 1D-CNN used the learning abilities of neural networks to identify by stacking fully connected layers and using one-dimensional convolution, which helped reduce inter-symbol interference and minor nonlinear issues. The 2D-CNN changed the complex signal into a two-dimensional format that included both real and imaginary parts, allowing it to handle the amplitude and phase information of I/Q components at the same time.

## 4. Results and Discussions

To evaluate and examine the performance of the 2D-CNN, 1D-CNN, 1D-DNN, and VNE, the three neural network models in the experiment utilized identical parameter configurations and employed the same training and testing datasets. Table 2 presents the pertinent parameter values.

The training approach used 100 epochs and a batch size of 512. These parameters were established through numerous trials, ensuring both training efficacy and enhanced training efficiency. The network used a two-layer design: the first layer had a 3 × 2 convolutional kernel (time span × real-imaginary channels), where the three-symbol coverage helped track the phase consistency between nearby symbols, and the two channels referred to the in-phase (I) and quadrature-phase (Q) parts of the QPSK signal. This setup allowed for simultaneous modeling of how the signal changed over time and the complex relationships between I/Q components. The second layer had a smaller 3 × 1 kernel, which kept the focus on capturing important time-related features while also looking for more complex patterns. This design allowed the network to simultaneously understand how the signal changes over time and how the I/Q components were related to each other in a complex way. The second-layer kernel was reduced to 3 × 1, which kept the ability to extract time-related features while focusing on capturing more complex abstract features. The pooling layer implemented (2, 1) subsampling to reduce feature dimensions along the time axis, hence augmenting translation invariance while maintaining complete information in the I/Q channels. An L1 regularization term ($\lambda =10^{-6}$) was added to the loss function to help prevent overfitting by encouraging the weight matrix to have fewer non-zero values. We included a Dropout layer at the network output with a probability of 0.2, which randomly deactivated neurons, thereby enhancing the ability to generalize. A dynamic early stopping mechanism (patience = 25) monitored validation loss and halted training when performance remained unchanged for 25 consecutive epochs, therefore preventing overfitting and optimizing computing resource use. We empirically confirmed that a batch size of 512 optimized CPU memory limitations and gradient update stability, facilitating reliable training convergence in intricate channel settings.

The design of the VNE detailed in this work specifies the tap count of the LMS linear filter as 15 and the order of the Volterra nonlinear filter as 13. The settings were adjusted through testing: the 13th-order Volterra structure found a good balance between handling nonlinearity and being efficient in calculations, as improvements levelled off and the risk of overfitting rose if we went beyond this order. The 15-tap version of the LMS filter adequately addressed the principal inter-symbol interference (ISI) range and reduced unnecessary calculations, as established by the autocorrelation analysis of the received signals. Dynamic step-size adjustment ($u_{LMS\_init}=2\times 10^{-5},\;u_{volterra}=2\times 10^{-6}$) and a regularization parameter ($\lambda_{init}=10^{-6}$) were used to make the model more stable and adaptable, helping to effectively correct for nonlinear distortions and phase noise in long-distance transmissions.

**Figure 4:**
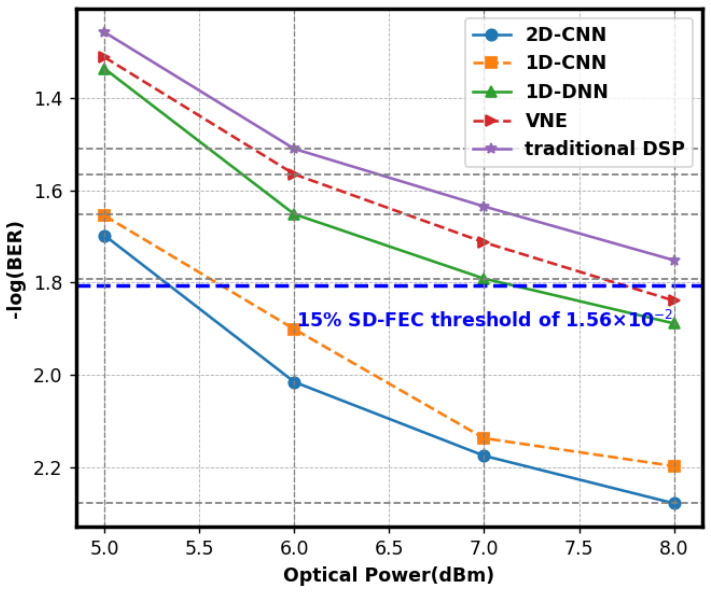
Curve of the BER performance for 2D-CNN, 1D-CNN, 1D-DNN, and VNE.

Figure 4 illustrates the BER curves with optical power into PD. The figure illustrates that, under varying optical power settings, the 2D-CNN exhibited the lowest overall BER and optimal performance. At an optical power of 8 dBm, the BER was 5.3 × 10^−3^, which was below the threshold for soft-decision forward error correction of 1.56 × 10^−2^ with a 15% overhead. It could precisely process communication signals and diminish the error rate. The BER of the 1D-CNN was marginally superior to that of the 2D-CNN, varying between 2.2 × 10^−2^ and 6.3 × 10^−3^. Due to its processing of one-dimensional data, it inadequately leveraged the correlation of two-dimensional spatial signals, leading to marginally diminished performance. The BER of the 1D-DNN ranged from 1.3 × 10^−2^ to 4.6 × 10^−2^. The structure was susceptible to overfitting, resulting in inadequate generalization capability and a comparatively elevated BER. The conventional VNE approach exhibited the greatest BER, ranging from 1.4 × 10^−2^ to 4.9 × 10^−2^. It possessed constraints in addressing intricate issues, and its efficacy was subpar compared to neural networks.

**Figure 5:**
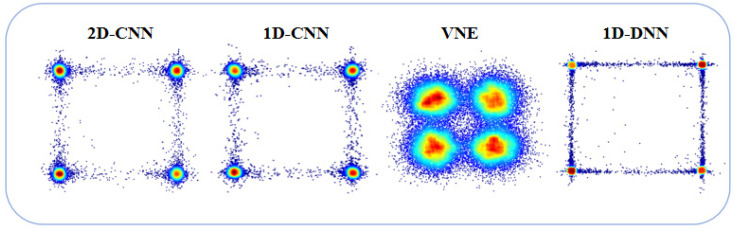
Comparison of signal constellation diagrams for various neural networks and VNE (optical power: 8 dBm).

We looked at the constellation diagrams of the VNE, 1D-DNN, 1D-CNN, and 2D-CNN to better understand how well they communicate and process signals. The constellation diagram illustrates signal quality and interference resilience. Figure 5 shows that the traditional VNE’s constellation diagram had a lot of spread and unclear points, meaning it struggled to reduce nonlinear distortion and noise interference, resulting in poor signal quality and a higher BER. The 1D-DNN constellation diagram exhibited dispersion and indistinct points, indicating overfitting and insufficient adaptability. The 1D-CNN constellation points were spread out and not grouped together well, which shows that it struggled to pick out important features and can’t effectively handle interference from nonlinearity and phase noise, possibly leading to signal distortion and a higher BER. In contrast, the 2D-CNN constellation diagram exhibited closely clustered points with little dispersion, facilitating separation. The figure shows that the 2D-CNN could adjust phase settings using convolutional network weights to rebuild the phase with very few errors. The constellations focused on standard QPSK constellation points, minimizing nonlinear and phase noise interference. The more concentrated and prominent constellation points exhibited superior signal quality and resilience to interference.

**Figure 6:**
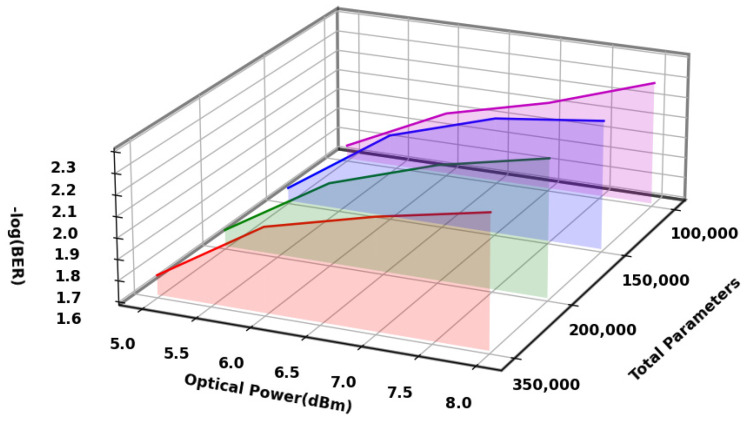
BER performance curve of 2D-CNN with varying quantities of trainable parameters.

To see how the number of trainable parameters affects the performance of 2D-CNN, we ran tests with four different amounts of trainable parameters: 100,000, 150,000, 200,000, and 350,000. During the tests, we kept everything the same, including the network design, activation function (ReLU), loss function (MSE), optimizer (AdamW), convolution kernel size, pooling method, and the datasets used for training and testing, with the only change being the number of trainable parameters. As shown in Figure 6, increasing the number of trainable parameters from 100,000 to 150,000 lowered the system’s BER from 5.9 × 10^−3^ to 5.6 × 10^−3^, which means that adding a moderate number of parameters helped the model learn complex signal details better and improved its ability to handle nonlinearity and phase noise. As the parameters were elevated to 200,000 and 350,000, the BER climbed marginally to 6.0 × 10^−3^ and 6.3 × 10^−3^, respectively, indicating a tendency of minor performance deterioration.

**Figure 7:**
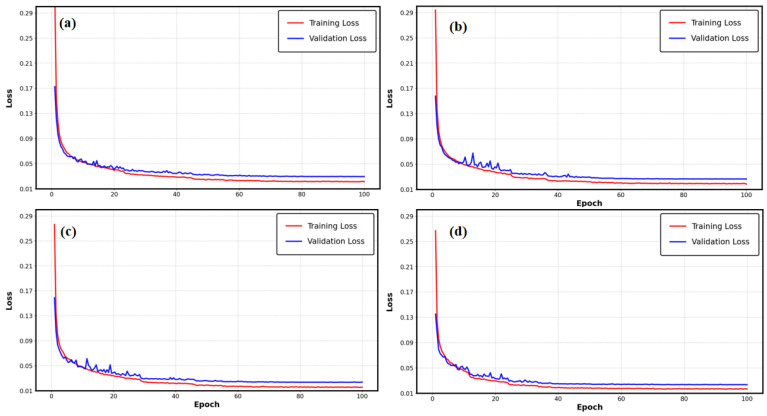
Training and validation loss curves for different numbers of trainable parameters at 8 dBm. (**a**) 100,000 parameters; (**b**) 150,000 parameters; (**c**) 200,000 parameters; (**d**) 350,000 parameters.

The training and validation loss curves at 8 dBm optical power for various parameter configurations in Figure 7a–d indicate that the losses for all four groups consistently decreased with the increase in epochs, while the disparity between training and validation losses remained minimal. The training and validation loss graphs for the four sets of parameters show that there was no sign of overfitting; all graphs show a steady decrease during training, with the final values staying close together and no major splits observed. This result was mostly due to the weight-decay regularization technique used by the AdamW optimizer, which substantially reduced the danger of overfitting as the parameter scale expanded. When the quantity of trainable parameters surpassed 150,000, the decline in BER optimization performance was not due to overfitting; rather, it was likely a consequence of redundant feature representation or the introduction of extraneous noise from an excess of trainable parameters, which increased the model’s sensitivity to dynamic channel fluctuations. Experimental results showed that the 150,000-parameter setup struck the best balance between being complex enough and being able to generalize well: it reduced the chance of underfitting by effectively extracting features (which was shown by the higher BER in the 100,000-parameter setup) while avoiding issues of wasted computing power and increased noise that come with having too many parameters.

**Figure 8:**
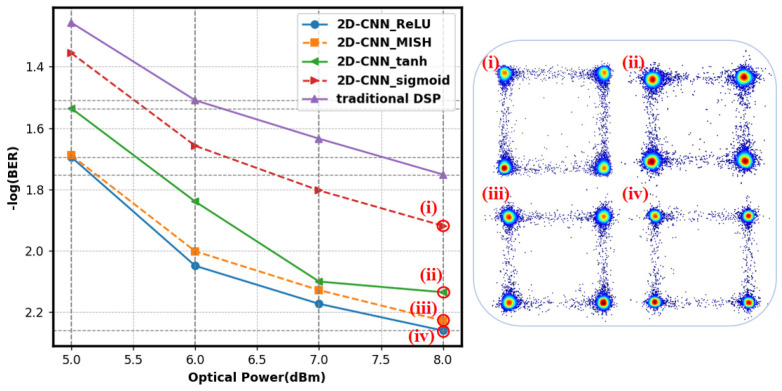
Impact of various activation functions on the BER of 2D-CNN, with insets of recovered constellation diagrams for (**i**) Sigmoid, (**ii**) Tanh, (**iii**) Mish, and (**iv**) ReLU with an optical power of 8 dBm.

Figure 8 compares CNN BER with different activation functions. The figure shows that the 2D-CNN with ReLU activation function had the lowest BER of 5.5 × 10^−3^, suggesting higher performance. The main reasons ReLU works are ReLU has an easy mathematical expression. This function saves computational power and speeds model training. Second, the ReLU function handles gradient vanishing well. Vanishing gradients hamper deep neural network model training. When x > 0, the ReLU function gradient was 1, avoiding gradient vanishing and improving model convergence. The Sigmoid function outputted 0–1. The gradient approached zero when the input value was extremely high or low, increasing BER and preventing parameter updates. The Tanh function reduced gradient vanishing, but its output was limited to −1 and 1; thus, minor gradients still affected training. Mish had a greater BER than ReLU since its properties did not match the 2D-CNN model’s tasks, leading to lower performance. Therefore, in this experiment, the ReLU function outperformed Mish as an activation function.

**Figure 9:**
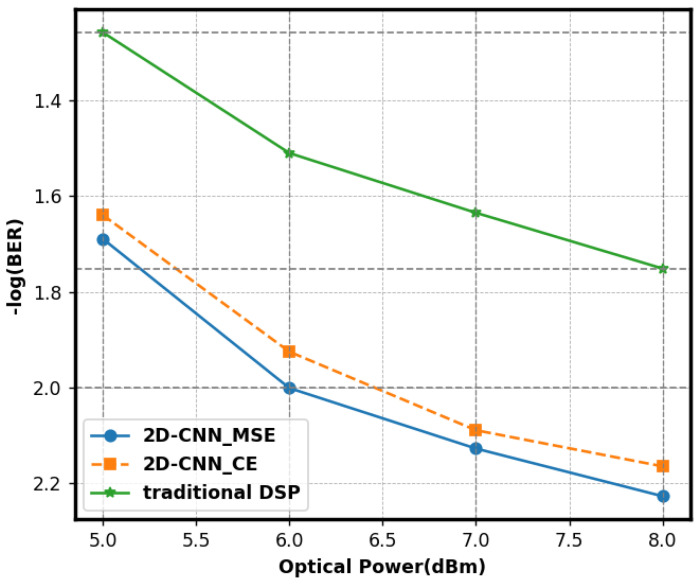
The BER performance of the 2D-CNN utilizing various loss functions.

Figure 9 illustrates the BER curves of the 2D-CNN using MSE and cross-entropy (CE) loss functions. We regulated variables and maintained other factors at a steady level. Utilizing MSE as the loss function, the 2D-CNN achieved a minimal BER of 5.9 × 10^−3^, signifying outstanding performance. Regression problems were addressed with MSE. The signal-processing environment of this experiment rendered signal prediction a regression problem. The MSE quantified deviation by averaging the squared discrepancies between expected and actual values. BER was significantly reduced during training since it emphasized absolute error and systematically adjusted parameters to align predicted values with actual values. The cross-entropy loss function measured the discrepancies across probability distributions in classification tasks. The regression of communication signals employed an error assessment method that failed to satisfy operational requirements, resulting in an increased BER. In short, the MSE loss function improved how well the model worked and reduced the BER in 2D-CNN communication signal processing.

**Figure 10:**
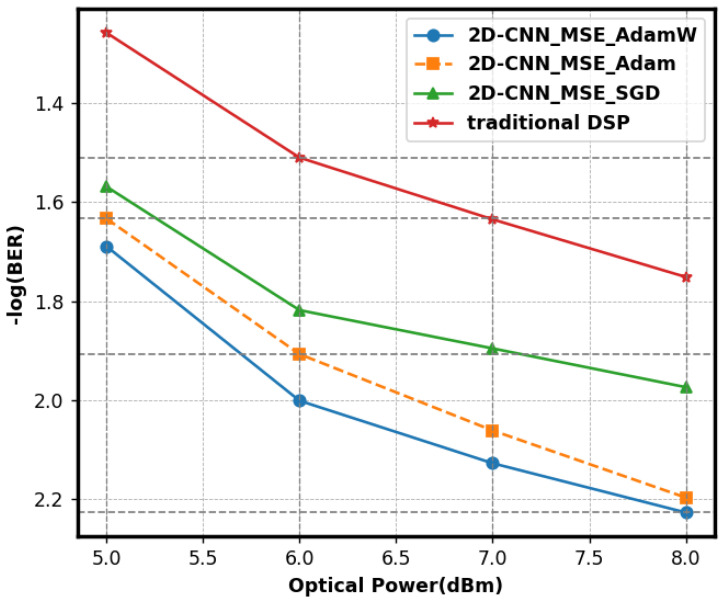
The impact of various optimizers on the BER of 2D-CNN.

We used the widely used optimizers AdamW, Adam, and SGD to evaluate their impact on the performance of 2D-CNNs. Figure 10 illustrates the BER comparisons across several optimizers. The AdamW optimizer trained the 2D-CNN, achieving a minimum BER of 5.9 × 10^−3^, signifying optimal performance. AdamW mitigated model overfitting with a weight-decay method derived from Adam. It may adjust the learning rate for each parameter and dynamically modify the update step size based on the gradient and historical gradients to expedite convergence to the optimal solution while maintaining stability. Adam, without a weight-decay mechanism, exhibited overfitting with intricate data and had a high BER. SGD employed a fixed learning rate and updated step magnitude. Large datasets and complex models made it hard to reach the best solution and could become stuck in less optimal points, leading to a higher BER.

## 5. Conclusions

This research has successfully demonstrated a long-distance wireless communication system using photonics-aided technology, allowing for a stable transmission of 50 Gbps (25 Gbaud) QPSK signals over 4600 m at a carrier frequency of 128.75 GHz. At an optical power of 8 dBm, the BER reached 5.3 × 10^−3^, which is lower than the threshold of 1.56 × 10^−2^ for SD-FEC with a 15% overhead. A method using a 2D-CNN which allows for the simultaneous correction of phase noise and nonlinear distortion was proposed to equalize nonlinear impairments and phase noise interference in the RoF system. Compared to traditional Volterra series and DNN methods, it has shown significant improvements, providing a new and effective way to fix signal problems in high-speed wireless communication systems. It offers essential technological assistance for the development of future 6G and higher-speed, high-capacity wireless communication networks.

## Figures and Tables

**Table 1:** Terahertz signal transmission performance with various equalizers and modulations.

Number	Frequency	Equalizer	Modulation Format	Distance	BER
[18]	120 GHz	2D-CNN	16-QAM	200 m	≤3.8 × 10^−3^
[19]	125/135/145 GHz	Volterra equalizer	PDM-16-QAM	4600 m	≤2.4 × 10^−2^
[20]	110–170 GHz	LSTM + RF	QPSK	20 km	<3.8 × 10^−3^
[21]	340 GHz	2D-CNN	16-QAM	54.6 m	≤10^−2^
[22]	125 GHz	LSTM + RF	16-QAM	4.5 m	5.64 × 10^−4^
[23]	286/299/312 GHz	MMA + Viterbi	64-QAM	30 m	<4.5 × 10^−3^

**Table 2:** Parameter values in 2D-CNN, 1D-CNN, and 1D-DNN.

Term	1D-DNN	1D-CNN	2D-CNN
Layers	3 FCL	2 Conv + 2 FCL	2 Conv (3 × 2, 3 × 1) + 2 FCL
Kernel size	Not used	(3), (3)	(3, 2), (3, 1)
Activation	ReLU	ReLU	ReLU
Epoch	100	100	100
Batch Size	512	512	512
Loss function	MSELoss	MSELoss + L1	MSELoss + L1
Optimizer	AdamW	AdamW	AdamW
Pooling size	Not used	(2)	(2, 1)
Total Parameters	239,042	139,802	139,702
MACC	236,160	229,440	229,440

## Data Availability

The raw/processed data required to reproduce these findings cannot be shared at this time as the data also form part of an ongoing study.
